# Prevention of Vomiting after Inhalation of Chloroform.—Fischer

**Published:** 1861-06

**Authors:** L. Elsberg


ACCORDING TO DR. FISCHER, a glass of wine taken 15–30 minutes before chloroform, will entirely prevent the vomiting so often troublesome after inhalation. Perhaps, he adds, the dangers may also be lessened by this simple means. [We feel bound to confirm Dr. F.'s positive statement to some extent, having frequently with great benefit administered wine before producing anæsthesia. The desired effect seemed in all cases to be produced more quickly and safely, (*i. e.*, with a smaller quantity of the anæsthetic,) and though we had not directed special attention to the vomiting afterwards, we do not remember that it ensued in a single one of these cases. Our experience in this respect is further borne out by that of a friend, a dentist, of this city.]—*Dr. L. Elsberg, Foreign News Compiler, American Medical Monthly.*

